# Extracting laboratory test information from paper-based reports

**DOI:** 10.1186/s12911-023-02346-6

**Published:** 2023-11-06

**Authors:** Ming-Wei Ma, Xian-Shu Gao, Ze-Yu Zhang, Shi-Yu Shang, Ling Jin, Pei-Lin Liu, Feng Lv, Wei Ni, Yu-Chen Han, Hui Zong

**Affiliations:** 1 Department of Radiation Oncology, Peking University First Hospital, No.7 Xishiku Street, Beijing, 100034 China; 2 Philips Research China, Shanghai, 200072 China

**Keywords:** Laboratory test, Paper based medical reports, Optical character recognition, Information extraction, Conditional random fields

## Abstract

**Background:**

In the healthcare domain today, despite the substantial adoption of electronic health information systems, a significant proportion of medical reports still exist in paper-based formats. As a result, there is a significant demand for the digitization of information from these paper-based reports. However, the digitization of paper-based laboratory reports into a structured data format can be challenging due to their non-standard layouts, which includes various data types such as text, numeric values, reference ranges, and units. Therefore, it is crucial to develop a highly scalable and lightweight technique that can effectively identify and extract information from laboratory test reports and convert them into a structured data format for downstream tasks.

**Methods:**

We developed an end-to-end Natural Language Processing (NLP)-based pipeline for extracting information from paper-based laboratory test reports. Our pipeline consists of two main modules: an optical character recognition (OCR) module and an information extraction (IE) module. The OCR module is applied to locate and identify text from scanned laboratory test reports using state-of-the-art OCR algorithms. The IE module is then used to extract meaningful information from the OCR results to form digitalized tables of the test reports. The IE module consists of five sub-modules, which are time detection, headline position, line normalization, Named Entity Recognition (NER) with a Conditional Random Fields (CRF)-based method, and step detection for multi-column. Finally, we evaluated the performance of the proposed pipeline on 153 laboratory test reports collected from Peking University First Hospital (PKU1).

**Results:**

In the OCR module, we evaluate the accuracy of text detection and recognition results at three different levels and achieved an averaged accuracy of 0.93. In the IE module, we extracted four laboratory test entities, including test item name, test result, test unit, and reference value range. The overall F1 score is 0.86 on the 153 laboratory test reports collected from PKU1. With a single CPU, the average inference time of each report is only 0.78 s.

**Conclusion:**

In this study, we developed a practical lightweight pipeline to digitalize and extract information from paper-based laboratory test reports in diverse types and with different layouts that can be adopted in real clinical environments with the lowest possible computing resources requirements. The high evaluation performance on the real-world hospital dataset validated the feasibility of the proposed pipeline.

## Introduction

Electronic medical records (EMRs), also known as computerized patient record, have been widely applied during patient assessment, examination, diagnosis, and treatment planning in many hospitals and healthcare centers around the world [1]. In China, the average adoption rate of EMRs in hospitals increased by 3.6 times from 2007 to 2018, peaking at 85.3% [2]. This has led to an unprecedented accumulation of medical data. According to the EMR-related health industry standards issued by China in 2016, there are a total of 53 types of EMRs. Efficient information extraction and knowledge mining from EMRs can facilitate translational medicine research and the development of clinical support system [3–5].

With the development and improvement of medical-related technologies, clinical auxiliary examinations are playing an increasingly important role in the diagnosis and treatment of diseases [6]. Laboratory tests, often part of routine checkups, aim to monitor patient condition by testing samples of blood, urine, or other body specimens [7]. As a result, a large number of reports with various types are produced, such as liver function test reports, blood test reports, urine test reports, genetic test reports, drug concentration test reports, etc. Laboratory test reports are stored and managed using Laboratory Information Management System (LIS). They can be delivered to patient in printed paper format or transmitted to the Hospital Information System (HIS) to assist physician for diagnosis [8].

Despite the substantial adoption of these electronic health information systems, a significant proportion of these reports still exist in paper-based formats, posing challenges for efficient data extraction and analysis. Health information exchange and patient engagement have remained limited [9]. In hospital setting, it is not uncommon for patients to carry lots of paper-based laboratory test reports for physicians to review. Physicians have to sift through the patient’s previous laboratory test reports from other hospitals, combining them with the patient’s chief complaint to form an initial diagnostic impression. During follow-up visits, the paper-based laboratory test reports are scanned and uploaded to specific application, requiring physicians to review these images and manually enter the lab test results into a database. The current processing of paper-based reports heavily relies on manual operations, resulting in time-consuming, labor-intensive and costly outcomes.

To address these problems, various artificial intelligence techniques can be utilized to automate the current manual processing of paper-based reports. Optical Character Recognition (OCR) is a technology that enables the conversion of scanned or photographed images of text into machine-readable and editable text. Deep-learning based OCR algorithm have been validated for extracting text information from scanned image of hardcopy medical reports [10, 11]. However, due to the different quality of the original hardcopy of the reports, the extracted text results from OCR algorithm often have recognition errors and cannot accurately preserve the original text layout information. Therefore, it is not viable to digitize paper-based reports and make them appropriate for database entry only based on the OCR results; further information extraction techniques are required.

Information extraction (IE) is the process of automatically extracting structured and meaningful information from unstructured or semi-structured data sources to enable further analysis and utilization of the extracted data. Information extraction from laboratory test results can be challenging due to their diverse non-standard layouts and mixed data types including text, numeric values, reference ranges, datetime and units [12]. To extract meaningful information from the OCR results of laboratory test reports, we need to identify and classify multiple named entities from the reports into predefined categories, such as report datetimes, names of the test item, test values and units. The task is called Named Entity Recognition (NER) in the field of Natural Language Processing (NLP). Conditional Random Fields (CRF) is a widely used NLP algorithm in NER [13], known for its ability to model the dependencies between neighboring words and leverage contextual information for accurate entity recognition [14]. Therefore, it is possible to cope with laboratory test reports with different layouts and mixed data type.

In this study, we developed a practical lightweight pipeline to digitalize and extract information from paper-based laboratory test reports that can be adopted in real clinical environments with the lowest possible computing resources requirements. The proposed pipeline consists of two modules: the Optical Character Recognition (OCR) module and the Information Extraction (IE) module. The OCR module is used to convert scanned image of paper-based laboratory test reports into semi-structured text items. In the IE module, the laboratory test reports time and test results are extracted using Conditional Random Fields (CRF)-based approach. The extracted test results are represented as tables including four types of named entities: test item name (LabName), test result (LabResult), unit (LabUnit) and reference value range (LabRefRange). Given an image of paper-based laboratory test report, the proposed pipeline is able to automatically output the report time and a digitalized table containing four columns, including LabName, LabResult, LabUnit and LabRefRange, which can be easily adapted for follow-up interoperation and reuse. We evaluated the performance of both the OCR module and the IE module using the dataset collected from Peking University First Hospital (PKU1). With an average accuracy of 0.93 for the OCR module, and an F1 score of 0.86 for the IE module, the performance of the proposed pipeline can meet the requirements of physicians in their daily work. We also validated the feasibility of the proposed pipeline in real hospital setting with a self-developed web-based application with the assistant of physicians at Peking University First Hospital. To our knowledge, no study has validated the feasibility and performance of such pipelines in a hospital setting with end-to-end applications used by physicians. By applying the proposed pipeline to automate the information extraction process from paper-based laboratory test reports, healthcare organizations can reduce manual work, eliminate human errors, and accelerate data utilization, eventually leading to better patient care and clinical decision-making.

The rest of the paper is organized as follows. The related work reviews previous studies on OCR and various downstream tasks related to paper-based medical documents. The methods section details the data description, data annotation and the proposed lightweight pipeline, incorporating an OCR module and an IE module based on CRF techniques, as well as the evaluation methods of the OCR module and the IE module respectively. The results section shows the implementation details and evaluation results of the OCR module and the IE module. The discussion section presents the principal findings, limitations, and future research. The conclusion summarizes the study, emphasizing the feasibility of the proposed lightweight pipeline.

## Related work

To extracting meaningful information from paper-based medical documents, researchers typically follow a two-step process. The first step involves utilizing OCR to extract text items from scanned or photographed documents. The second step involves applying information extraction techniques to accomplish downstream tasks. OCR algorithms have been extensively studied and developed to convert images to text items. State-of-the-art deep learning-based OCR algorithms have shown high detection accuracy in a wide range of application scenarios [15–17]. For medical documents such as electronic health records (EHRs) and medical lab reports, OCR algorithms have also demonstrated a high level of detection accuracy ranging from 78.84% to 95.8% [18, 19].

Once the text has been extracted using OCR, a range of downstream tasks have been accomplished using various Natural Language Processing (NLP) and deep learning approaches. Goodrum et al. [20] combined OCR with multiple text classification models to classify scanned EHRs into clinically relevant and non-clinically relevant categories, as well as further sub-classifications. Kumar et al. [21] developed a ClinicalBERT-based pipeline to identify malpractice claims from scanned mammograms, chest CT and bone X-ray reports following the OCR step. Wei et al. [22] implemented a multi-modal system that jointly models text extracted from OCR and layout information to classify scanned clinical documents into different categories, such as lab reports and CT scans. Hsu et al. [11] proposed a pipeline to classify scanned sleep study reports and found that the ClinicalBERT-based approach obtained the best classification accuracy performance.

Previous studies have put the major efforts on improving the accuracy of the downstream task, and large-scale language models such as BERT [23], and ClinicalBERT [24] have been widely adopted. However, deploying these large-scale models in real-world application suffers from the problems of high hardware resources requirements (e.g., high-performance GPUs) and long inference time. With a single Intel Xeon Gold 6154 CPU @ 3.00 GHz, the average inference time of large-scale BERT-based methods (with 110 million parameters) ranges from 4.43 s to 18.66 s per sample for different datasets and NLP tasks [25]. To overcome these problems, our proposed pipeline focused on balancing the accuracy performance and the inference time while avoiding additional hardware expenses for hospitals that adopt such a system.

## Methods

**Fig. 1:**
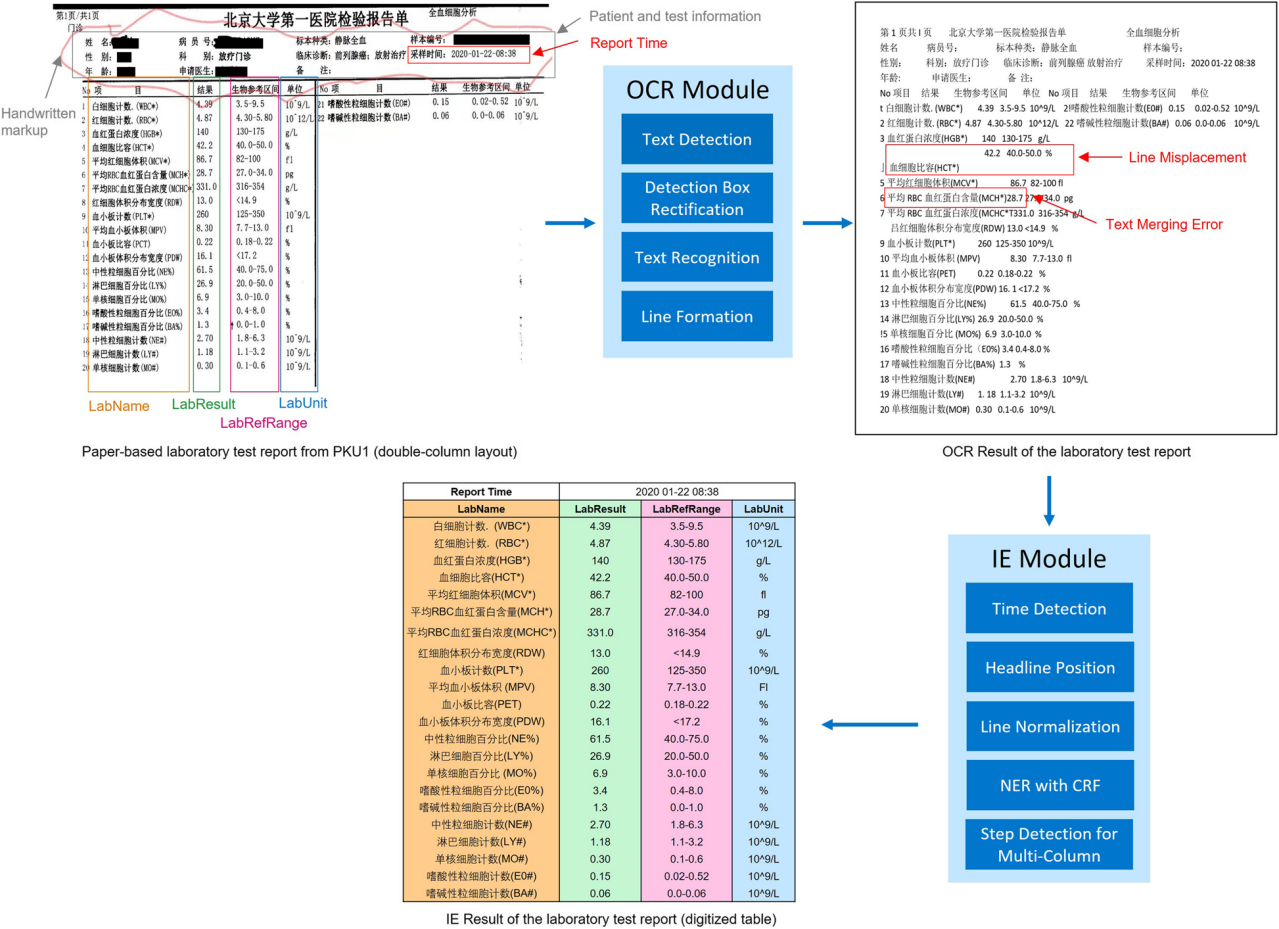
Overall illustration of the pipeline with the optical character recognition (OCR) module and the information digitization (IE) module for digitization of paper-based laboratory test reports

The proposed information digitalization and extraction pipeline is illustrated in Fig. 1. Given a scanned image of paper-based laboratory test report as input, the text detector in the OCR module detects the locations of textual objects in the mage using detection boxes. Then, the text recognizer identifies the content of these textual objects, and the line formation formats the textual objects into lines. The output of the OCR module is a collection of text items that partially retains the original formatting, which may contain recognition errors as marked in the figure, such as the text merging errors (two textual objects merged as one text item) and misplaced lines. Given the output of the OCR module as input, the IE module extracts and recognizes the report time and four types of named entities in laboratory tests: test item name (LabName), test result (LabResult), unit (LabUnit) and reference value range (LabRefRange). After the IE module, the final output of the pipeline is the digitalized table of test results including four columns: LabName, LabResult, LabUnit, and LabRefRange. The digitalized table can be easily restored in a database and used for downstream tasks.

### Data description

We used medical laboratory reports from an open-source dataset for the development of the OCR module and the training of the IE module [18]. The dataset contains 238 de-identified images of Chinese medical laboratory reports. These images are captured by different devices (i.e., scanners and smart phones) and under various illuminant conditions from 119 paper files of Chinese medical laboratory reports. Each report contains four parts arranged from top to bottom: 1) report time and test type 2) patient and test information 3) the table of test results including four columns: test item name, test result, unit, and reference value range 4) signature and notes.

We also collected a total of 196 real-world laboratory test reports from Peking University First Hospital (PKU1) for performance validation. Reports with incomplete scanning and very low image quality (where the recognition accuracy of OCR is lower than 30%) were excluded, resulting in 153 laboratory reports in the final dataset. These reports are used to evaluate the performance of OCR module and IE module, respectively. These laboratory test reports are coming from different hospitals brought by patients visiting PKU1 from other hospitals. There are also various types of laboratory test reports. The detailed report types and the amounts of each type are shown in Table 1. Therefore, the collected laboratory rest reports have diverse layouts. Ethics approval was granted by the Ethics Committee of Peking University First Hospital.

**Table 1:** The laboratory test report types and amounts in the PKU1 dataset

Test Report Type	Test Report Amount
Complete blood cell count	49
Prostate-specific antigen (PSA) test	27
Biochemical test	26
Tumor marker test	12
Urinalysis (urine test)	9
Viral test	7
Other	23
Total	153

### Data annotation

**Fig. 2:**
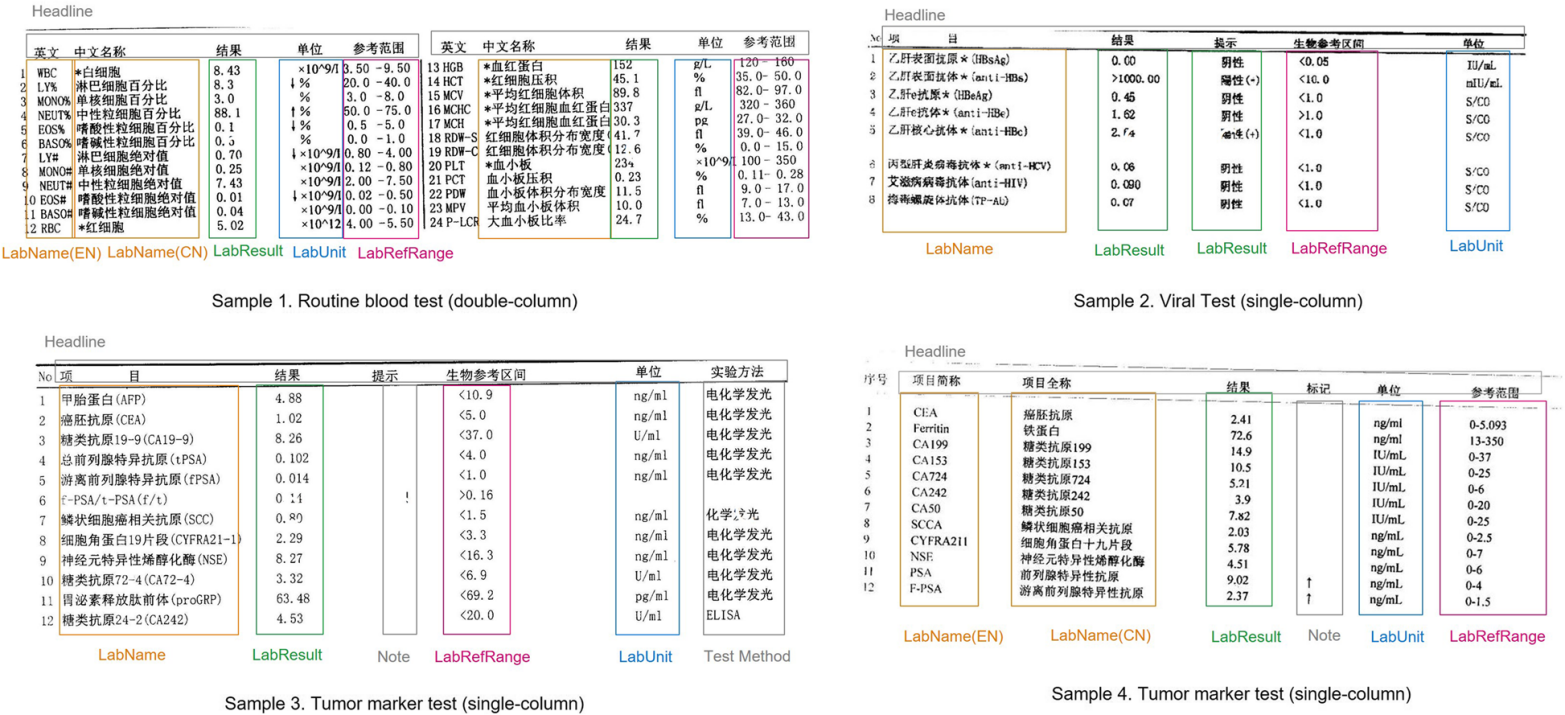
Sample laboratory test reports from Peking University First Hospital (**1**) sample 1: complete blood cell count (**2**) sample 2: viral test (**3**) sample 3: tumor marker test (**4**) sample 4: tumor marker test

Since the paper-based laboratory test reports vary in types and originate from different hospitals, there is no standard format. To obtain the final digitalized table with four columns, as illustrated in Fig. 1, we need to annotate the starting and ending position of each text item and categorize it into one of the four categories: LabName (test item name), LabResult (test result), LabUnit (unit), and LabRefRange (reference value range). Figure 2 displays four sample test reports with different layouts and the categories of text items (the starting and ending positions of text items are also annotated but not illustrated in the figure). Sample 1 has a double-column layout, requiring annotation for both columns. Sample 1 and sample 4 have both English LabName and Chinese LabName. Both LabNames are annotated and can be detected and linked as a single LabName in the IE module. Sample 2 caontians two LabResults, one indicating the test value and the other indicating whether the result is positive or negative. Similarly, both LabResults are annotated and can be detected and linked as a single LabResult in the IE module. Furthermore, the order of the four categories can vary among samples. For example, the order of LabUnit and LabRefRange differs between sample 3 and sample 4. There may be additional columns beyond the four categories, such as the test method in sample 3 and notes in sample 3 and sample 4. These columns are not annotated and will not be detected and recognized by the IE module. Apart from the four categories, we also annotated the report time in the laboratory reports. When there are multiple datetime items, we used the earliest datetime value as the report time. The starting and ending position are represented in the IOB (Inside, Outside, Beginning) format for Named Entity Recognition (NER) task. For example, in a text sequence with six Chinese characters, if the first four characters form a LabName, the first character is labeled as B-LabName (beginning of a LabName), the second to fourth characters are labeled as I-LabName (inside a LabName), and the fifth and sixth characters are labeled as O (outside a named entity).

We developed a web-based annotation system. The frontend was a questionnaire webpage created using Pyramid, a lightweight Python web framework. The backend was developed to store data and annotations using MongoDB. The annotators were trained for the annotation task based on the annotation guidelines. Each report was annotated by two human annotators. Cohen’s kappa coefficient was calculated to measure the inter-rater reliability between annotators and high agreement ($\kappa =0.89$) was obtained.

### Optical Character Recognition (OCR)

Optical Character Recognition (OCR) is a widely used technology to recognize texts within images. In our system, we adopted the state-of-the-art OCR model, PP-OCR [16], to extract text from scanned images of paper-based reports from hospitals. The key advantage of PP-OCR is its ultra-lightweight nature. With only 3.5 M size model, the PP-OCR system can recognize 6622 Chinese characters and a 2.8 M size model to recognize 63 alphanumeric symbols. Moreover, the average inference time is 421 ms using a single CPU. PP-OCR consists of three modules: text detection, detection boxes rectification and text recognition. The text detection module aims to locate the text area in the image. In PP-OCR, a simple segmentation network called Differentiable Binarization (DB) [17] is used as the text detector. The detection boxes rectification module transforms the detected text boxes with different directions into horizontal rectangle boxes. A text direction classifier is trained to determine the direction of the detected text box, and geometric transformation is then applied. The text recognition module is used to recognize the text within the detection box. For text recognition, an end-to-end scene text recognition model named CRNN (Convolutional Recurrent Neural Network [15]) is adopted. To reduce the model size of detection boxes rectification module and text recognition module, MobileNet V3 [26] is employed as the lightweight backbone.

Following the preceding procedures, the scanned images are transformed into text items with detection boxes. The final step of the OCR module is the Line Formation, which organizes the text items into lines based on the coordinates of individual detection boxes. Text items with similar vertical coordinates are grouped together and classified as belonging to the same line. Consequently, the OCR module generates formatted lines of text items that can by readily utilized by the IE module.

### Information extraction (IE)

**Fig. 3:**
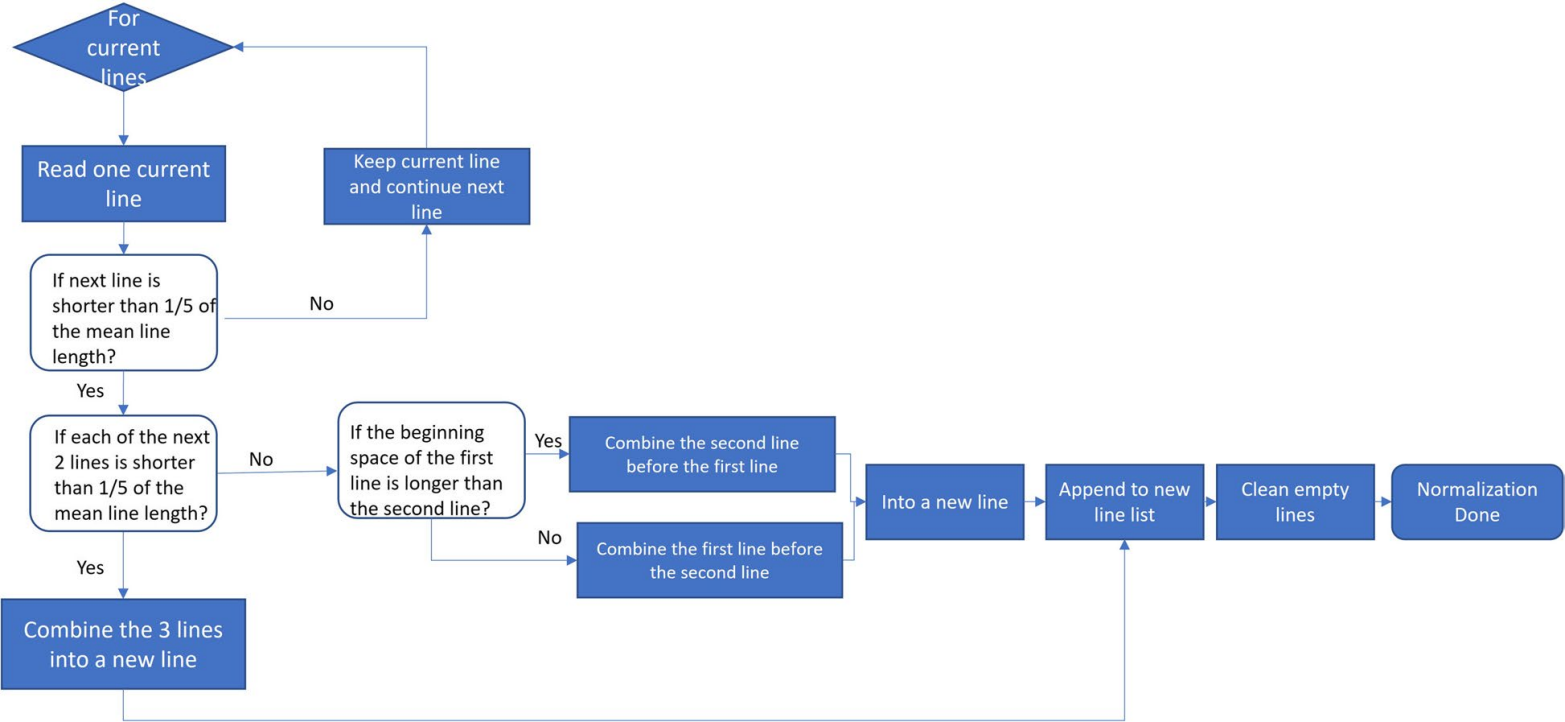
Decision diagram of the Line Normalization algorithm

Although the OCR module has recognized the text items and retained partial structural information, it cannot be directly transformed into a digitalized table for use in downstream tasks. There three main challenges that need to be addressed. Firstly, the semantic meaning of each text item is not recognized, requiring Named Entity Recognition (NER) algorithms to classify each text item into the predefined four categories (LabName, LabResult, LabUnit, and LabRefRange). Secondly, the OCR outcomes usually contain recognition errors, such as the text merging error (two textual objects merged as one item) and misplaced lines, as illustrated in Fig. 1. To tackle the text merging error, NER algorithms were employed to identify the correct starting and ending potion for each text item. To address the misplaced lines, we implemented the Line Normalization algorithm. Lastly, we developed the Step Detection algorithm to handle reports with a multi-column layout. In summary, there are five sub-modules in the IE module: Time Detection, Headline Position, Line Normalization, NER with CRF, and Step Detection for Multi-column. The implementation details of each sub-module are as follows:

- 1) Time Detection: It detects all time elements and selects the earliest time as the report time.
- 2) Headline Position: It identifies both the head and tail lines to facilitate the location of the recognition range for the NER task. Regular expressions containing keywords are utilized to match headline and endline. For example, keyword “name” is used to identify the headline of LabName, while “result” or “value” is used to identify the headline for LabResult.
- 3) Line Normalization [27]: The mean length of all lines in a report (denoted as $\overline{L }$) is calculated. The text is processed line by line, where the line in processing is designated as the “current line.” The line length of the next line or the next two lines is then compared to the mean line length $\overline{L }$ following the rules outlined in Fig. 3. This process determines whether and how these lines should be combined into one new line. Finally, all empty lines are removed to complete line normalization.
- 4) NER with CRF: The data is processed in a row-wise manner, and predictions are generated using a Conditional Random Fields (CRF) model [28] trained on the aforementioned public dataset (as described in the data description section). As mentioned in the data annotation section, all text items are annotated with the beginning and ending positions, as well as the categories. Feature engineering was performed by incorporating word identity, word suffix, word shape, and part-of-speech tags. The CRF model was trained using the L-BFGS [29] training algorithm with Elastic Net (L1 + L2) [30] regularization on the annotated training set. Once the CRF model is trained, given an input report, it can predict the starting position, ending position and category (LabName, LabResult, LabUnit, LabRefRange) of each text item in the report.
- 5) Step Detection for Multi-column: After performing NER using the CRF model, step detection is conducted for each line of the report. The basic concept of the Step Detection algorithm is that once the second LabName is detected, the report is identified as having a double-column layout. The starting position of the second LabName serves as the reference for splitting the repeated columns. Similarly, if the third LabName is detected, the report can be identified as having tripe-column layout. Post-processing is also applied to ensure that the content in multi-column layout is correctly placed in the single-column digitalized table, which serves as the final outcome of the entire IE module.

### Evaluation

Aligned with the design of model pipeline, the evaluation of the model encompasses two main components: the evaluation of the OCR module and the evaluation of the IE module.

#### Evaluation methods of OCR results

As described in the OCR module methodology, the OCR module process involves the conversion of scanned images into text items and detection boxes, followed by the transformation of the text into formatted lines. Therefore, we used three accuracy measurements to measure the performance in different aspects, which are character level accuracy, detection box level accuracy, and line formation accuracy.

Character accuracy is employed to assess the correctness of individual characters recognized by the OCR module. For instance, if there are 100 characters in the scanned images and 90 of them are correctly identified, the character level accuracy would be calculated as 90%. Subsequently, the detection box level accuracy focuses on evaluating the accuracy of the detection boxes, without considering character accuracy. For instance, if the report consists of 10 detection boxes, but two of them are incorrectly recognized as one, it indicates that two detection boxes were not accurately identified. In this case, the detection box level accuracy would be 80%. Lastly, considering the character and detection box inaccuracies addressed by the aforementioned accuracy metrics, the line formation accuracy is utilized to measure the correctness of the formatted lines. For example, if there are 10 lines of text in the scanned images, and one line is mistakenly recognized as two separate lines, the number of correctly formatted lines would be 9, resulting in a line formation accuracy of 90%. The final accuracy of the OCR module is defined as the average of the above three accuracies, providing an overall assessment of its performance.

#### Evaluation methods of IE results

We evaluate the results of IE module using precision (the number of correctly recognized entities divided by the number of all recognized entities), recall (the number of correctly recognized entities divided by the number of all annotated entities in data), and F1 score (the harmonic mean of precision and recall). When both the text content and category match the ground truth, it is considered as a correctly recognized entity. All recognized entities are all text items identified and classified by the IE module. All annotated entities are all text items annotated by human annotators. It is noteworthy that LabName can exist in both Chinese and English. When the Chinese LabName is correct, the LabName is considered as correctly recognized.

## Results

### Implementation details

The proposed pipeline was implemented in Python 3.8, utilizing PaddlePaddle for OCR modeling, scikit-learn 0.23.2 for CRF modeling, and nltk 3.6.1 for word segmentation and POS tagging. The self-developed pipeline application was executed on a Linux operating system (Ubuntu 18.04 LTS), leveraging the computational capabilities of an Intel Xeon Silver 4116 CPU operating at a frequency of 2.10 GHz, with a total RAM capacity of 32 GB.

### Performance of OCR module

The evaluation was conducted using real-world laboratory test reports collected from PKU1. A total of 153 scanned images of reports were used encompassing various types as descripted in the data description section. As indicated in the evaluation methodology for the OCR module, the assessment of OCR performance included three key metrics: character level accuracy, detection box level accuracy, and line formation accuracy. The detailed evaluation results are presented in Table 2. The average character level accuracy was determined to be 0.95, indicating a high precision in the recognition of individual text items by the OCR module. With regards to the detection box level accuracy, an average accuracy of 0.93 was obtained. It should be noted that in some cases, text present in separate columns might be erroneously recognized as a single detection box. Furthermore, 90% of the line formation process yielded accurate results. However, it is important to acknowledge that some reports exhibited varying degrees of skew, which presented challenges during the line formation step. Consequently, due to the presence of skewness, there were instances where one line was incorrectly treated as two separate lines.

**Table 2:** The evaluation results of OCR module

	**Character Level Accuracy**	**Detection Box Level Accuracy**	**Line Formation Accuracy**	**Overall**
Average accuracy	0.95	0.93	0.90	0.93

### Performance of information extraction

To establish a baseline for comparing the CRF-based NER models, we implemented a rule-based model. Precisely, the rule-based model consists of custom regular expressions developed based on existing reports. For example, the "LabName" entity mainly comprises Chinese characters, while the "LabResult" entity mostly consists of unsigned numbers or Chinese characters (such as 'negative'), except for the period ".". The "LabRefRange" entity includes symbols such as " < ", " > ", or "-", along with numbers and Chinese characters. The "LabUnit" entity exhibits combinations that include "/", "%", or English characters. Additionally, we defined rules to predict the category of the header based on keywords. The category of header can then be used to predict the categories of text items below the header.

We assessed the performance of the IE module using 153 paper-based laboratory reports collected from PKU1, as described in the data description section. These reports exhibited diverse checking items and inconsistent layouts, reflecting the diversity and complexity of laboratory examinations in real clinical practice. We compared the rule-base method and the CRF-based method on the dataset. As shown in Table 3, the CRF-based method outperformed the rule-based method, achieving an overall F1 score of 0.86 with a precision of 0.90 and recall 0f 0.83. More specific results using the CRF-based method for different entity categories are shown in Table 4. It can be found that the performance of LabName, LabResult, and LabUnit surpassed that of the LabRefRange.

**Table 3:** Information extraction performance comparison of rule-based method and CRF-based model

	**Results for Rule-based**	**Results for CRF-based**
Precision	0.84	0.90
Recall	0.73	0.83
F1	0.78	0.86

**Table 4:** Precision, recall, and F1 scores for each type of data elements of reports from PKU1

	**Lab Name**	**Lab Result**	**Lab Ref Range**	**Lab Unit**	**Overall**
Correctly predicted Entities	1862	1786	1620	1697	6965
Entities in the gold standard	2140	2140	2115	2047	8442
Precision	0.91	0.92	0.84	0.85	0.90
Recall	0.87	0.83	0.77	0.83	0.83
F1	0.89	0.87	0.80	0.84	0.86

### Error analysis

**Fig. 4:**
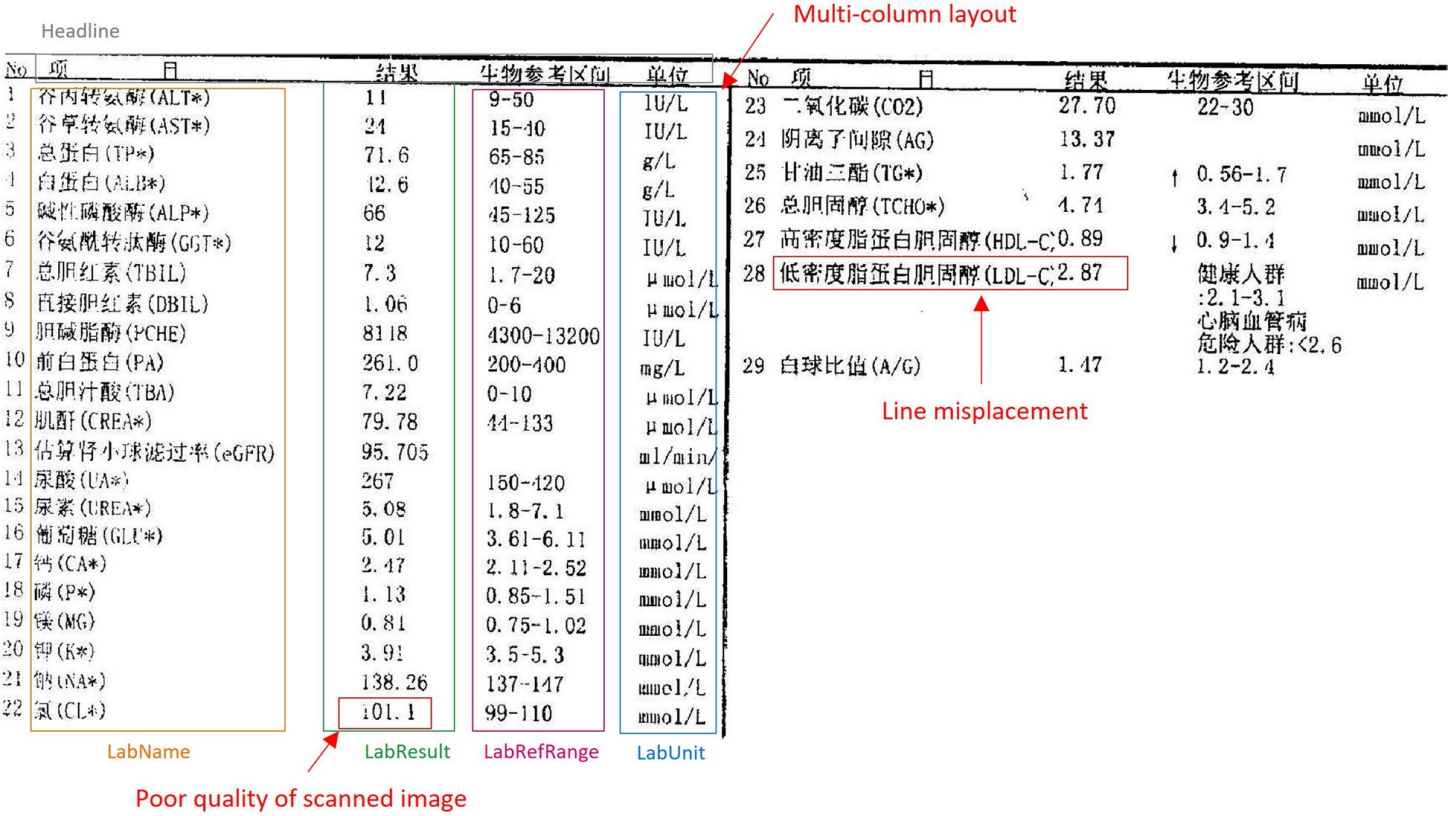
Demonstration example of a laboratory test report of two-columns layout from Peking University First Hospital. Different laboratory test entities are marked by distinct colors with category names noted. The red box represents the factors contributing to the recognition errors

Based on our analysis, there are three main factors contributing to recognition errors: 1) poor quality of the scanned image, 2) line misplacement, and 3) multi-column layout. Figure 4 shows a typical sample report that exhibits all three factors and results in low recognition performance (with an average precision of 0.59). Due to the poor quality of scanned image, LabResult “101.1” was recognized as “01.1” in the final IE result. As shown in Fig. 4, the misplacement of lines caused the test value “2.87” to be associated with test item No. 27 “(HDL-C)” instead of the correct test item No. 28 “(LDL-C)”. Furthermore, the multi-column layout let to the incorrect recognition of test item No. 29 “(A/G)”.

## Discussion

### Principal findings

In this study, we implemented an information extraction and digitization pipeline to automatically retrieve laboratory test time and test results (i.e., LabName, LabResult, LabUnit and LabRefRange), from paper-based laboratory test reports. With an averaged recognition accuracy of 0.93 for the OCR module, and the F1 score of 0.86 for the IE module, the results demonstrated the effectiveness of the proposed pipeline in extracting test results accurately. Moreover, the proposed pipeline showed high runtime efficiency. With a single Intel Xeon Silver 4116 CPU, the average inference time of each report was only 0.78 s which is significantly faster than the inference time of BERT-based models (4.43 s to 18.66 s) [25]. We also developed an end-to-end web-based application, which has been used by physicians at Peking University First Hospital in real hospital environments. This validation further confirmed the feasibility of the proposed pipeline and demonstrated the significant benefits of the pipeline for physicians.

### Limitations

The major limitation of the proposed pipeline is that the accuracy of the OCR module and IE module can be affected by the quality of the scanned image, including issues such as image clarity, image completeness, and handwritten markups, which are not uncommon in clinical practice. These factors may introduce errors and reduce the overall performance of the proposed pipeline and require manual efforts from physicians to revise the pipeline outputs.

Another limitation of the proposed pipeline is the adoption of a CRF-based information extraction method to improve overall runtime efficiency, instead of using advanced BERT-based methods. However, the recent emergence of lightweight and small-scale BERT models, such as TinyBERT [31], MobileBERT [32] and ALBERT [33], offers a possible future direction to resolve the paradox of the pipeline accuracy and efficiency.

### Future research

This study specifically focuses on the digitalization of paper-based laboratory test reports. However, in clinical practice, patients often present a variety type of paper-based reports, such as pathology reports, radiology reports, admission summaries, and discharge summaries. To fully digitize and extract information from all types of medical records, there is a pressing need to extend the current pipeline to encompass a broader range of paper-based medical records. In our ongoing study, we have made initial attempts to apply our proposed pipeline to radiology reports from computed tomography (CT) and magnetic resonance imaging (MRI).

## Conclusion

In this study, we developed an information digitization pipeline for scanned images of laboratory test reports in diverse types and with different layouts. The pipeline demonstrated high recognition accuracy, runtime efficiency, and real-world applicability. It holds significant potential to streamline and enhance information extraction in clinical practice.

## Data Availability

The open-source dataset analyzed during the current study is available in the repository https://github.com/xuewenyuan/OCR-for-Medical-Laboratory-Reports. The PKU1 datasets generated during the current study are available from the corresponding author on reasonable request.

## Abbreviations

EMRs: Electronic medical records
LIS: Laboratory information management system
HIS: Hospital information system
NLP: Nature language processing
OCR: Optical character recognition
CRNN: Convolutional recurrent neural network
CRF: Conditional random fields
NER: Named entity recognition
PKU1: Peking University First Hospital

## References

[1] Adler-Milstein J, DesRoches CM, Kralovec P, Foster G, Worzala C, Charles D (2015). Electronic health record adoption in US hospitals: Progress continues, but challenges persist. Health Aff (Millwood).

[2] Liang J, Li Y, Zhang Z, Shen D, Xu J, Zheng X (2021). Adoption of electronic health records (EHRs) in China during the past 10 years: consecutive survey data analysis and comparison of sino-american challenges and experiences. J Med Internet Res.

[3] Ford E, Carroll JA, Smith HE, Scott D, Cassell JA (2016). Extracting information from the text of electronic medical records to improve case detection: a systematic review. J Am Med Inform Assoc.

[4] Wang Y, Wang L, Rastegar-Mojarad M, Moon S, Shen F, Afzal N (2018). Clinical information extraction applications: a literature review. J Biomed Inform.

[5] Cai T, Zhang L, Yang N, Kumamaru KK, Rybicki FJ, Cai T (2019). EXTraction of EMR numerical data: an efficient and generalizable tool to EXTEND clinical research. BMC Med Inform Decis Mak.

[6] Mishra N, Duke J, Karki S, Choi M, Riley M, Ilatovskiy AV (2021). A modified public health automated case event reporting platform for enhancing electronic laboratory reports with clinical data: design and implementation study. J Med Internet Res.

[7] Dikmen ZG, Pinar A, Akbiyik F (2015). Specimen rejection in laboratory medicine: necessary for patient safety?. Biochem Med (Zagreb).

[8] Pylypchuk Y, Meyerhoefer CD, Encinosa W, Searcy T (2022). The role of electronic health record developers in hospital patient sharing. J Am Med Inform Assoc.

[9] Furukawa MF, King J, Patel V, Hsiao C-J, Adler-Milstein J, Jha AK (2014). Despite substantial progress in EHR adoption, health information exchange and patient engagement remain low in office settings. Health Aff (Millwood).

[10] Laique SN, Hayat U, Sarvepalli S, Vaughn B, Ibrahim M, McMichael J (2021). Application of optical character recognition with natural language processing for large-scale quality metric data extraction in colonoscopy reports. Gastrointest Endosc.

[11] Hsu E, Malagaris I, Kuo Y-F, Sultana R, Roberts K (2022). Deep learning-based NLP data pipeline for EHR-scanned document information extraction. JAMIA Open..

[12] Cassim N, Mapundu M, Olago V, Celik T, George JA, Glencross DK (2021). Using text mining techniques to extract prostate cancer predictive information (Gleason score) from semi-structured narrative laboratory reports in the Gauteng province, South Africa. BMC Med Inform Decis Mak.

[13] Liu P, Guo Y, Wang F, Li G (2022). Chinese named entity recognition: the state of the art. Neurocomputing.

[14] Lafferty J, McCallum A, Pereira FC. Conditional random fields: probabilistic models for segmenting and labeling sequence data. 2001.

[15] Shi B, Bai X, Yao C (2016). An end-to-end trainable neural network for image-based sequence recognition and its application to scene text recognition. IEEE Trans Pattern Anal Mach Intell.

[16] Du Y, Li C, Guo R, Yin X, Liu W, Zhou J, et al. PP-OCR: A practical ultra lightweight OCR system. 2020.

[17] Liao M, Wan Z, Yao C, Chen K, Bai X. Real-time scene text detection with differentiable binarization. 2019. 10.1109/TPAMI.2022.3155612 35239474

[18] Xue W, Li Q, Xue Q (2020). Text detection and recognition for images of medical laboratory reports with a deep learning approach. IEEE Access.

[19] Batra P, Phalnikar N, Kurmi D, Tembhurne J, Sahare P, Diwan T. OCR-MRD: performance analysis of different optical character recognition engines for medical report digitization.

[20] Goodrum H, Roberts K, Bernstam EV (2020). Automatic classification of scanned electronic health record documents. Int J Med Informatics.

[21] Kumar A, Goodrum H, Kim A, Stender C, Roberts K, Bernstam EV (2022). Closing the loop: automatically identifying abnormal imaging results in scanned documents. J Am Med Inform Assoc.

[22] Wei Q, Zuo X, Anjum O, Hu Y, Denlinger R, Bernstam EV, Citardi MJ, Xu H. ClinicalLayoutLM: a pre-trained multi-modal model for understanding scanned document in electronic health records. In 2022 IEEE International Conference on Big Data (Big Data) 2022;2821–2827). IEEE.

[23] Devlin J, Chang MW, Lee K, Toutanova K. Bert: Pre-training of deep bidirectional transformers for language understanding. arXiv preprint arXiv:1810.04805. 2018.

[24] Alsentzer E, Murphy JR, Boag W, Weng WH, Jin D, Naumann T, McDermott M. Publicly available clinical BERT embeddings. arXiv preprint arXiv:1904.03323. 2019.

[25] Chen L, Varoquaux G, Suchanek FM (2021). A lightweight neural model for biomedical entity linking. Proc AAAI Confer Artif Intell.

[26] Howard A, Sandler M, Chu G, Chen L-C, Chen B, Tan M, et al. Searching for MobileNetV3. 2019.

[27] Rashid SF, Shafait F, Breuel TM. Scanning neural network for text line recognition. In: 2012 10th IAPR International Workshop on document analysis systems. 2012. p. 105–9.

[28] Zheng H, Qin B, Xu M. Chinese Medical Named Entity Recognition using CRF-MT-Adapt and NER-MRC. In: 2021 2nd International Conference on Computing and Data Science (CDS). 2021. p. 362–5.

[29] Zhu C, Byrd RH, Lu P, Nocedal J (1997). Algorithm 778: L-BFGS-B: Fortran subroutines for large-scale bound-constrained optimization. ACM Trans Math Softw.

[30] Haynos AF, Wang SB, Lipson S, Peterson CB, Mitchell JE, Halmi KA (2021). Machine learning enhances prediction of illness course: a longitudinal study in eating disorders. Psychol Med..

[31] Jiao X, Yin Y, Shang L, Jiang X, Chen X, Li L, Wang F, Liu Q. Tinybert: Distilling bert for natural language understanding. arXiv preprint arXiv:1909.10351. 2019.

[32] Sun Z, Yu H, Song X, Liu R, Yang Y, Zhou D. Mobilebert: a compact task-agnostic bert for resource-limited devices. arXiv preprint arXiv:2004.02984. 2020.

[33] Lan Z, Chen M, Goodman S, Gimpel K, Sharma P, Soricut R. Albert: A lite bert for self-supervised learning of language representations. arXiv preprint arXiv:1909.11942. 2019.

